# The Impact of Perceptions of Positive COVID-19 Information on Travel Motivation and Intention: Evidence From Chinese University Students

**DOI:** 10.3389/fpsyg.2022.871330

**Published:** 2022-03-31

**Authors:** Shanshan Li, Chenyu Liu, Zhusheng Wu, Ying Ma, Baoxia Chen, Shiying Gao, Zichao Chen, Shuang Xin

**Affiliations:** ^1^School of Physical Education, Sichuan University, Chengdu, China; ^2^Department of MA Filmmaking, University for the Creative Arts, Farnham, United Kingdom; ^3^State Information Center, Beijing, China; ^4^School of Tourism and Urban-rural Planning, Zhejiang Gongshang University, Hangzhou, China

**Keywords:** COVID-19 pandemic, travel intention, theory of planned behavior, travel motivation, university students

## Abstract

The COVID-19 pandemic has influenced the tourism industry in various ways, including tourists’ travel motivations and intentions. Unlike previous studies that have focused on the dark side of the pandemic, this study adds the dimension of perceptions of positive information on COVID-19 to the Theory of Planned Behavior to explore their influence on travel motivation and intention. A total of 470 valid questionnaires were collected from a sample of Chinese university students. The results showed that the students’ perceptions of positive COVID-19 information positively impacted their travel intentions through the variables of perceived behavioral control, travel attitudes, and travel motivations. Perceived behavioral control was the mediating variable that most explained the impact of perceptions of positive COVID-19 information on travel motivation and intention. This study contributes to the understanding of the influence of the COVID-19 pandemic on tourism and of university students’ travel motivations and intentions. It also offers implications for the tourism industry to formulate relevant recovery strategies during and after the pandemic.

## Introduction

The novel coronavirus (COVID-19) pandemic has been deemed the most influential and destructive event of the 21st century, especially for the tourism industry (WTTC, 2020). It led many countries and regions to impose travel restrictions, which has had a serious impact on the tourism industry (Yang et al., 2020). As a result of the pandemic, 96% of European countries enforced travel bans, and the valuation of hotels, airlines, and cruise companies declined significantly (Peter and Dritan, 2020; Sharma and Nicolau, 2020). According to the United Nations World Tourism Organization, one in three destinations worldwide were closed (UNWTO, 2021b), and the arrivals of international tourists was decreased by 72% from January 2020 to December 2020 (UNWTO, 2021a). Due to this, the export revenues have a loss of USD 935 billion (Kumar et al., 2021). Meanwhile, the risk of infection has become a major health concern that affects travel intention (Jonas et al., 2010). According to Richter (2003), it is important to recognize the threat of global public health to tourism, which can lead to great uncertainty in people’s future travel intentions and motivations. The most significant related concern centers on the inhibition of tourism demand and the obstacles to tourists’ travel planning decisions as a result of the outbreak of infectious diseases (Kuo et al., 2008).

One of the most popular research topics has been the extensive impact of the pandemic on the tourist behavior, including travel intention and motivation (Sánchez-Cañizares et al., 2021). Unlike previous studies that have focused on the dark side of the pandemic, this study explores the impact of people’s perceptions of positive information related to COVID-19 on their travel motivations and intentions. The positive information of COVID-19 refers to the information that indicates the positive changes of the pandemic situation, including news regarding declining death and infection rates, news about successful vaccination drives, news of re-openings of transportations, news of re-openings of visitor attractions, and so on.

This study selected one significant tourism market segment in China, Chinese university students, as its research object. University students’ travel intentions and motivations have attracted previous scholarly attention (Kim et al., 2012). Deng and Ritchie (2018) classified the risks perceived by university students during traveling into human-made, social-psychological, financial, and health categories. When these risks are perceived to be increasing in the tourism destination, students’ travel intentions decrease due to health and safety concerns (Hartjes et al., 2009). However, on the other hand, university students are young and “allocentric” therefore tending to be risk-takers and crisis-resistant tourists (Hajibaba et al., 2015). Do university students’ risk perceptions of the COVID-19 pandemic impact their travel motivations and intentions? If the information perception is positive, will they be willing to travel? This study aims to explore these questions.

As the first country to report cases of COVID-19, China was selected as the research context. Since the outbreak of COVID-19, information about the epidemic has infiltrated the daily life of the Chinese. Apart from the complete closure of China’s tourism industry during the initial stage, the principle of limited and orderly opening has been adopted since April 8, 2020, after the pandemic was gradually controlled. Meanwhile, Chinese university students have been forced to stay at home for a long time due to the lockdown that has been enforced. Their chances of travel have been reduced, and their travel motivation was suppressed. Consequently, they may be more eager to travel and pay more attention to information related to the pandemic, especially positive information. Thus, to understand whether university students are willing to travel during the pandemic, this study explores the impact of perceptions of positive COVID-19 information on travel intentions and motivations. Due to outbound travel has been restricted in China since the break of COVID-19 pandemic, travel in the present study refers to domestic travel only.

## Materials and Methods

### Research Design

A questionnaire was designed based on the literature and the background of the COVID-19 pandemic with five dimensions (see Table 1). The maximum variance method was used for exploratory factor analysis, and items with a factor load less than 0.6 were excluded. A seven-point Likert scale was adopted, in which 1 represented “strongly disagree” and 7 represented “strongly agree.”

**Table 1:** Research variables of the survey.

Variable	Indicator
Travel Attitude (Ragheb and Beard, 1982; Ramamonjiarivelo et al., 2015)	1. I think proper travel during the pandemic is acceptable. 2. I think travel is a good way to relax during the pandemic. 3. I think travel is a good form of entertainment during the pandemic. 4. I think travel is a good way to keep healthy during the pandemic. 5. I think travel is a good way to understand the real situation in society during the pandemic.
COVID-19 positive information perception	1. Tourism policy information during the pandemic would affect my participation in travel.
	2. The school’s relevant regulations during the pandemic would affect my participation in travel.
	3. The pandemic risk level at the destination would affect my participation in travel.
	4. The medical and health conditions at the destination would affect my participation in travel.
	5. The tourist traffic recovery during the pandemic would affect my participation in travel.
	6. The local tourism service during the pandemic would affect my participation in travel.
	7. Online public opinions during the pandemic would affect my participation in travel.
Tourism motivation (Lam and Hsu, 2006 Hsu and Huang, 2012)	1. I want to travel to ease the negative emotions brought about by the pandemic. 2. I want to travel to relieve the fatigue caused by the pandemic. 3. I want to travel to shop during the pandemic.
Perceived behavioral control (Han et al., 2010; Hsieh et al., 2016)	1. I have enough time to travel during the pandemic. 2. I have enough mental energy to travel during the pandemic. 3. I have enough physical energy to travel during the pandemic.
Travel intention (Zeithaml et al., 1996; Sparks, 2007)	1. I will travel after the ban (in my home city) is lifted. 2. I will travel after the ban (in the destination city) is lifted. 3. I want to travel after the risks posed by the pandemic become lower. 4. I will go to the open visitor attractions that are recommended by others during the pandemic.

We used the Questionnaire Star online questionnaire system^1^ to sample Chinese university students from November 20 to 30, 2020. The link of the online questionnaire was distributed to university students’ WeChat (the most popular social media in China) groups. A total of 470 effective questionnaires out of 500 were collected, with an effective recovery rate of 94%. The questionnaire included four descriptive questions on the students’ gender, grade, risk level in the region, and intended travel mode during the pandemic. SPSS 22.0 and AMOS 21.0 software were used for data analysis to explore the relationships among the various factors.

## Literature Review

### Travel Motivation

Scholars have divided the dimensions of travel motivation in different ways in the context of different theories. Yüksel et al. (2005) believed that escape, relaxation, enhancing mutual relationships, and self-realization and progress were tourists’ core motivations. According to Lee et al. (2004), motivations for tourism mainly include cultural attractions, family reunions, curiosity, attraction to local festivals, and the desire for emotional satisfaction. Travel motivations may vary among different tourists, especially those from different countries and cultural backgrounds (Kim and Prideaux, 2005). Travel motivation is also influenced by different values. People with internal values are more eager to visit new tourist sites, while people with external values are more concerned about the on-site experience and the excitement they gain from it (Li and Cai, 2012).

In the face of the sudden outbreak of a disease, people may decrease their travel motivations and concern about protecting themselves (Zheng et al., 2021). The COVID-19 pandemic has aroused people’s increased desire for safety (Rettie and Daniels, 2020), resulting in taking several measures to protect themselves, such as avoiding going out or visiting only places where there is little risk of infection (Zheng et al., 2021). Therefore, evading disease and danger may become a motivation for traveling (Kock et al., 2020). Qiao et al. (2021) asserted the relationship between the COVID-19 pandemic and tourists’ self-protection motivations. The present study seeks to investigate how people’s travel motivations are affected by their perceptions of positive COVID-19 information.

### Travel Intention

Travel intention has been reported to be influenced by a series of factors. Mohamad et al. (2012) claimed that a destination’s image can affect people’s willingness to visit it. Further, the promotion of destinations may positively affect people’s travel intention due to the lower costs (Jalilvand et al., 2012). Different personalities also lead to different travel intentions. Some people prefer familiar destinations, while others prefer those that are unfamiliar (Lepp and Gibson, 2008). Previous tourism experience is also considered to affect travel intention (Hsieh et al., 2016), and interest in the destination plays a significant role in travel intention and destination choice (Echtner and Ritchie, 1993).

Another factor that can influence travel intention is information. Most tourists use online information to increase their understanding of the destination before traveling (Narangajavana et al., 2017), and searching for information about the destination is considered a common process in tourism decision making (Amaro et al., 2016). Positive information, such as good E-WOM (electronic word-of-mouth), increases potential tourists’ travel intentions (Jalilvand et al., 2012). In contrast, negative information (e.g., information about risks) may decrease travel intentions (Beerli et al., 2007). In the context of the COVID-19 pandemic, tourists’ travel intentions are more related to the information about epidemic situation and related regulations of their destinations. The information available on the COVID-19 pandemic from different channels (e.g., news, social media, relatives, and friends) has tended to be extremely negative, especially in the early stage, and the emotional anxiety and fear caused by this information have led people to give up their travel plans (Bae and Chang, 2021).

The relationship between the COVID-19 pandemic and travel intention has been investigated by several studies. Luo and Lam (2020) found that fear of COVID-19 directly affects travel anxiety and risk attitude, which have direct negative effects on travel intention. Riestyaningrum et al. (2020) found that the pandemic had significant partial effects on international tourists’ travel intentions. Zenker et al. (2021) tested how tourists’ travel intentions are affected by their intra-personal anxiety. Zheng et al. (2021) asserted that travel intentions are influenced by tourists’ evaluation of the risks and coping strategies during the COVID-19 pandemic. However, these studies have tended to focus on the impact of negative information about the COVID-19 pandemic on travel intentions, while little attention has been paid to the possible positive changes to travel intention that may occur when the situation improves. Thus, to complement the existing literature, this study explores the influence of perceptions of positive COVID-19 information on travel intention.

### Information Perception

The influence of information perception on travel decisions has been confirmed by previous studies (Fan et al., 2018; Gössling et al., 2020). Before traveling, people search for relevant information about the destination in advance (Nunkoo et al., 2013). When people have a positive view of the information about a destination, their travel intention to this destination is stimulated (Woodside et al., 2011). However, when people perceive risks in a destination, the fear they feel further increases their self-protection motivations (Zheng et al., 2021). Once this perception of risks exceeds their psychological endurance, tourists may give up their travel plans altogether. This is consistent with the Basic Emotion Theory, which is central to the study of emotional expression, stating that emotions enable the individual to respond adaptively to evolutionarily significant threats and opportunities in the environment (Keltner et al., 2019).

Rittichainuwat and Chakraborty (2009) found that infectious diseases negatively impact tourists’ travel intentions due to the potential health risks they pose. The information on the associated risks will further determine whether people have enough driving force to travel. Tourists’ perceptions of risk information further aggravate the psychological barriers to travel to risky areas (Rittichainuwat and Chakraborty, 2009). The unique characteristics of COVID-19 have altered the risk perceptions associated with destinations (Jahari et al., 2021). Travelers’ risk perception predicts their information-seeking process, which helps them to accumulate the risk information that influences their travel intentions (Meng et al., 2021). Specifically, as a “misinfodemic” (Williams et al., 2020), the negative information portrayed in the mass media regarding the COVID-19 has increased people’s perceptions of the risks of traveling and their self-protection motivations (Qiao et al., 2021). Consequently, people have reduced their travel to protect their health during the pandemic (Yang et al., 2020).

However, that is not to say that people are unwilling to travel during the pandemic. People may generate positive travel intention even if they are in fear of COVID-19 (Yang et al., 2022). As Itani and Hollebeek (2021) revealed, visitors’ virtual reality tourism intentions increased when in-person travel was not feasible. Travel motivations and intentions that were suppressed by the pandemic will be liberated when the situation improves. The situation may also lead some people to pay special attention to the positive information related to the COVID-19 pandemic. According to Lu et al. (2021), people tended to seek COVID-19-related information from social media platforms where positive content was prevalent. They perceived these positive contents as desirable and necessary due to the positive impact on their emotions. Seeking further information is a common tourist strategy to reduce perceived risks (Reichel et al., 2009). According to Kuo et al. (2015), the perception of reliable, accurate, and easily available information can reduce the risks tourists perceive and stimulate their positive intentions to visit a destination. Furthermore, the public always trust and consider the information as reliable whenever it comes from the government (Mohammed et al., 2020) and their trust on the government could positively influence the risk perception during the pandemic situations (Tumlison et al., 2017). In the Middle East, tourists’ willingness to travel depends on their sense of security and government policies, as well as whether a vaccine against COVID-19 is available (Choufany, 2020). Ivanova et al. (2021) found that the health system and disinfection status of a destination also affect tourists’ destination choice. Qiao et al. (2021) investigated the role of positive mass media coverage in decreasing tourists’ self-protection motivations. Wang et al. (2021) found that during the COVID-19 pandemic, a low-risk perception has a positive influence on tourists’ attitude toward undertaking regional travel. The above studies have indicated that, as an “infodemic” (Williams et al., 2020), reliable and positive information on the COVID-19 pandemic may play a role in enhancing people’s travel intentions.

### The Theory of Planned Behavior in Tourism

According to the Theory of Planned Behavior, people’s behavior is not only determined by their will but also by their attitudes, subjective norms, and perceived behavioral control (Ajzen, 1991). This theory has been widely used in tourism research. Jalilvand et al. (2012) studied the influence of E-WOM on the choice of tourism destination by using the theoretical model of planned behavior. Sparks and Pan (2009) also revealed that subjective norms and perceived behavioral control were related to Chinese tourists’ choice of Australian destinations. Further, based on the Theory of Planned Behavior, Bamberg et al. (2003) investigated how tourists’ choice of travel mode is affected by their past behaviors, habits, and the information they have.

The application of the Theory of Planned Behavior to investigating travel intention has presented inconsistent findings. Lam and Hsu (2006) demonstrated that attitude and perceived behavioral control are factors that can effectively predict travel intention. Lee and Jan (2018) showed that environmental attitude, subjective norms, and perceived behavioral control have a positive impact on ecotourism intention. However, Sparks and Pan’s (2009) study suggested that the reasons behind different travel intentions may not be attributed to attitude and perceived behavioral control but rather to subjective norms. This indicates the necessity to test if the constructs of the Theory of Planned Behavior are effective in influencing people’s travel intentions during and after the COVID-19 pandemic. Sánchez-Cañizares et al. (2021) revealed the modulating effect of perceived risks on travel intention in the context of COVID-19 pandemic. Lucarelli et al. (2020) demonstrated that the construct relationships in the Theory of Planned Behavior were not weakened by the COVID-19 pandemic and that those who have good knowledge of COVID-19 and climate change exhibit higher pro-environmental behavioral intentions.

Specifically, Li et al. (2021) explored the significant changes in post-pandemic planned travel behaviors, finding that Chinese tourists’ travel intentions would still be negatively influenced even 6 months or longer after the COVID-19 pandemic was controlled. However, they did not take into account the quality and reliability of the information available, which may mediate the negative influence of perceptions of the COVID-19 pandemic on travel intention. Moreover, their research was conducted at the beginning of the COVID-19 outbreak (February 9, 2020) when people were overwhelmed by fears and worries during the initial quarantine. In contrast, the present study was conducted at the end of 2020, when the COVID-19 pandemic was under control in China and the country’s tourism industry had reopened. Contrary to the frightening information available in the early stage, the information available on the pandemic in China tended to be positive then, with few deaths and new cases of infection reported. With more knowledge and positive information about the COVID-19 pandemic, people’s travel intentions may be increased, and this study explores this issue in the framework of the Theory of Planned Behavior. However, we did not include the variable of subjective norms in our framework due to inadequate theoretical support in existing studies.

### Hypotheses

Since tourism is an information-intensive industry, accurate information input is an important factor for tourists’ destination choice (Lam and McKercher, 2013). Information on the current situation of the COVID-19 pandemic and the tourism industry can affect tourists’ personal cognition and subjective judgment. As previous studies have indicated (Reichel et al., 2009; Rittichainuwat and Chakraborty, 2009; Kuo et al., 2015), the more positive and reliable the information tourists receive, the fewer challenges and risks they perceive, and the more they perceive they can control of their travel behaviors. Therefore, we proposed the following hypothesis:

*Hypothesis 1 (H1)*: University students’ perceptions of the positive information on the COVID-19 pandemic have a positive impact on their perceived behavioral control of travel.

Han et al. (2010) observed that attitude is an important mediator between perceived behavioral control and behavioral intention. Perceived behavioral control also affects travel intention Hsu and Huang (2012). In addition, travel motivation is determined by tourists’ feelings and value system (Gnoth, 1997), which are also related to perceived behavioral control. The more tourists perceive they can control a situation, the stronger their travel motivation will be; thus, tourists’ willingness to travel to risky destinations is affected by how much they perceive they can control their behaviors in terms of the risks posed (Jonas et al., 2010). In this way, tourists traveling shorter distances from home or to familiar destinations would feel safer during the COVID-19 pandemic (Galoni et al., 2020) due to their stronger perceived behavioral control. Research has further shown that the risk of COVID-19 infection affects travel intention through perceived behavioral control (Sánchez-Cañizares et al., 2021). Therefore, this study proposed the following additional hypotheses:

*Hypothesis 2 (H2)*: University students’ perceived behavioral control has a positive impact on their travel attitudes during the COVID-19 pandemic.

*Hypothesis 3 (H3)*: University students’ perceived behavioral control has a positive impact on their travel intentions during the COVID-19 pandemic.

*Hypothesis 4 (H4)*: University students’ perceived behavioral control has a positive impact on their travel motivations during the COVID-19 pandemic.

As shown by Nunkoo et al. (2013), people’s travel intentions are related to their travel attitudes. When people have a positive attitude toward travel, they will also have positive travel intentions (Shen et al., 2019). Hsu and Huang (2012) also showed that travel motivation is an important predictor of travel intention. Therefore, Hypotheses 5 and 6 were proposed as follows:

*Hypothesis 5 (H5)*: University students’ travel attitudes have a positive impact on their travel intentions during the COVID-19 pandemic.

*Hypothesis 6 (H6)*: University students’ tourism motivations have a positive impact on their travel intentions during the COVID-19 pandemic.

Hsu and Huang (2012) verified that people’s motivation for travel affects their attitude toward it, while Mansour and Mumuni (2019) used the push-pull theory to demonstrate the impact of motivation on attitudes toward domestic tourism. Therefore, Hypothesis 7 was proposed as follows:

*Hypothesis 7 (H7)*: University students’ travel motivations have a positive impact on their travel attitudes during the COVID-19 pandemic.

**Figure 1:**
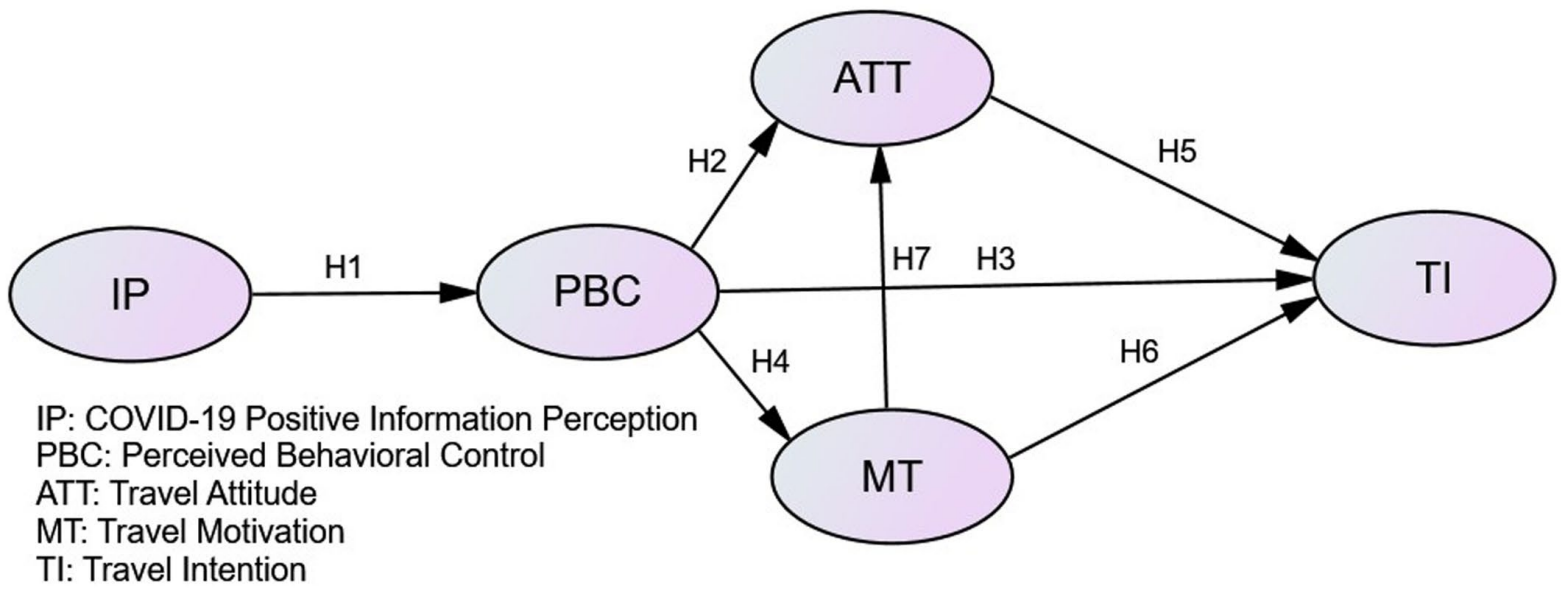
Conceptual model.

Based on the above hypotheses, a preset model was established, as shown in Figure 1.

## Results

### Demographic Information

The descriptive data are shown in Table 2. The sample comprised both male and female students, ranging from freshmen to postgraduate students. The location risk level is defined by the *New Coronavirus Pneumonia Prevention and Control Program* released by the National Health Committee of the People’s Republic of China. Specifically, low-risk regions: no confirmed cases or no new confirmed cases for 14 consecutive days; medium risk regions: there are new confirmed cases within 14 days, the cumulative confirmed cases are no more than 50, and there is no aggregated epidemic within 14 days; and high risk regions: there are more than 50 confirmed cases in total, and there is an aggregated epidemic situation within 14 days.

**Table 2:** Descriptive statistics (*N* = 470).

Measure	Item	Count	%	Measure	Item	Count	%
Gender	Male	214	45.5	Location risk level	Low risk	447	95.1
	Female	256	54.5		Medium risk	17	3.6
Grade	Freshman	59	12.6		High risk	6	1.3
	Sophomore	135	28.7	Preferred travel modes	Self-guided tour	236	50.2
	Junior	74	15.7		self-driving tour	192	40.9
	Senior	52	11.1		Package tour	36	7.7
	Master and above	150	31.9		A half package tour	6	1.3

### Reliability and Validity Analysis

Principal component analysis (PCA) was used to extract the questionnaire, and Caesar’s normal maximum variance method was used to rotate the questionnaire (Joreskog and Sorbom, 1989). The MT4 was deleted since the factor load in this scale was less than 0.60, and 22 valid questions were retained. The reliability and validity analysis showed that the factor load of each dimension was between 0.610 and 0.930, which met the requirements of the model.

The Cronbach’s α values ranged from 0.626 to 0.856, the component reliability ranged from 0.871 to 0.890, and the average variance extraction value ranged from 0.514 to 0.726 (Tables 3 and 4); thus, the reliability of all items met the requirements. The average variance extraction value (AVE) of each dimension was between 0.572 and 0.643. The parameter estimation of each measurement model and topic was significant, that is, *p* < 0.001. Therefore, the five subscales reached the ideal standard of reliability and aggregate validity.

**Table 3:** Measurement model.

Construct Research	Code item		Parameter significance estimate				Reliability of item	Composite reliability	Average variance extracted
			Unsted.	S.E.	Z-Value	P	std.	SMC	CR	AVE
ATT	ATT1	ATT	1				0.786	**0.618**	**0.890**	**0.670**
	ATT2	ATT	1.17	0.056	20.778	***	0.879	**0.773**		
	ATT4	ATT	1.193	0.06	19.735	***	0.841	**0.707**		
	ATT5	ATT	1.002	0.057	17.547	***	0.764	**0.584**		
IP	IP7	IP	1				0.606	**0.367**	**0.880**	**0.514**
	IP6	IP	1.172	0.087	13.432	***	0.809	**0.654**		
	IP5	IP	1.216	0.089	13.615	***	0.828	**0.686**		
	IP4	IP	1.103	0.088	12.503	***	0.727	**0.529**		
	IP3	IP	1.003	0.079	12.684	***	0.742	**0.551**		
	IP2	IP	0.964	0.088	11.008	***	0.611	**0.373**		
	IP1	IP	1.029	0.088	11.72	***	0.664	**0.441**		
MT	MT3	MT	1				0.639	**0.408**	**0.871**	**0.699**
	MT2	MT	1.454	0.091	16.027	***	0.925	**0.856**		
	MT1	MT	1.412	0.089	15.943	***	0.912	**0.832**		
PBC	PBC1	PBC	1				0.808	**0.653**	**0.888**	**0.726**
	PBC2	PBC	1.08	0.048	22.317	***	0.929	**0.863**		
	PBC3	PBC	0.905	0.046	19.87	***	0.814	**0.663**		
TI	TI1	TI	1				0.862	**0.743**	**0.880**	**0.648**
	TI2	TI	0.974	0.042	22.97	***	0.863	**0.745**		
	TI3	TI	0.794	0.043	18.646	***	0.747	**0.558**		
	TI4	TI	0.784	0.043	18.378	***	0.739	**0.546**		

*** *P* < 0.001 (the same below). Bold values of SMC, CR and AVE are corresponding to Squared Multiple Correlation, Construct Reliability and Average Variance Extracted.

**Table 4:** Discriminatory validity.

Dimension	Average variance extracted	Pearson correlation and discriminatory validity				
	AVE	MT	ATT	TI	PBC	IP
MT	0.648	**0.805**				
ATT	0.726	0.728	**0.852**			
TI	0.699	0.616	0.612	**0.836**		
PBC	0.670	0.448	0.485	0.573	**0.816**	
IP	0.514	0.247	0.194	0.124	0.257	**0.717**

Bold values on the diagonal are the square root values of the extracted average variance.

The square root of AVE was greater than the Pearson correlation coefficient between the dimensions below the diagonal, indicating each dimension had significant difference validity (Fornell and Larcker, 1981).

### Measurement Model Fit Test

According to Abd-El-Fattah (2010), the better the model fit, the closer the sample data and the model matrix. Both the absolute fit index and the relative fit index were used in the model fit test. The ratio of the chi-square value to the degree of freedom (*χ*^2^/df) eliminates the influence of the degree of freedom, and it is acceptable when less than 5. As shown in Table 5, the value of *χ*^2^/df was 3.471, the value of RMSEA was lower than 0.08. Although the values of GFI and AGFI were less than 0.9, they are close to 0.9 (0.848 and 0.880), which were also within the allowable range, as recommended by Hair et al. (1998). The relative fit indices usually include CFI, IFI, and NNFI, which were all higher than 0.9 in this study. Overall, this study’s model had a good fit.

**Table 5:** Index table of SEM model fitness.

Model fitting degree	Standard	Actual fitting degree of model	Model fitting judgment
*ML χ* ^2^	As small as possible	631.772	Reach standard
df	As small as possible	182	Reach standard
Normed Chi-sqr(*χ*^2^/df)	1 < *χ*^2^/df < 5	3.471	Reach standard
RMSEA	<0.08	0.073	Reach standard
TLI(NNFI)	>0.9	0.916	Reach standard
CFI	>0.9	0.927	Reach standard
IFI	>0.9	0.928	Reach standard

### Hypothesis Testing

**Figure 2:**
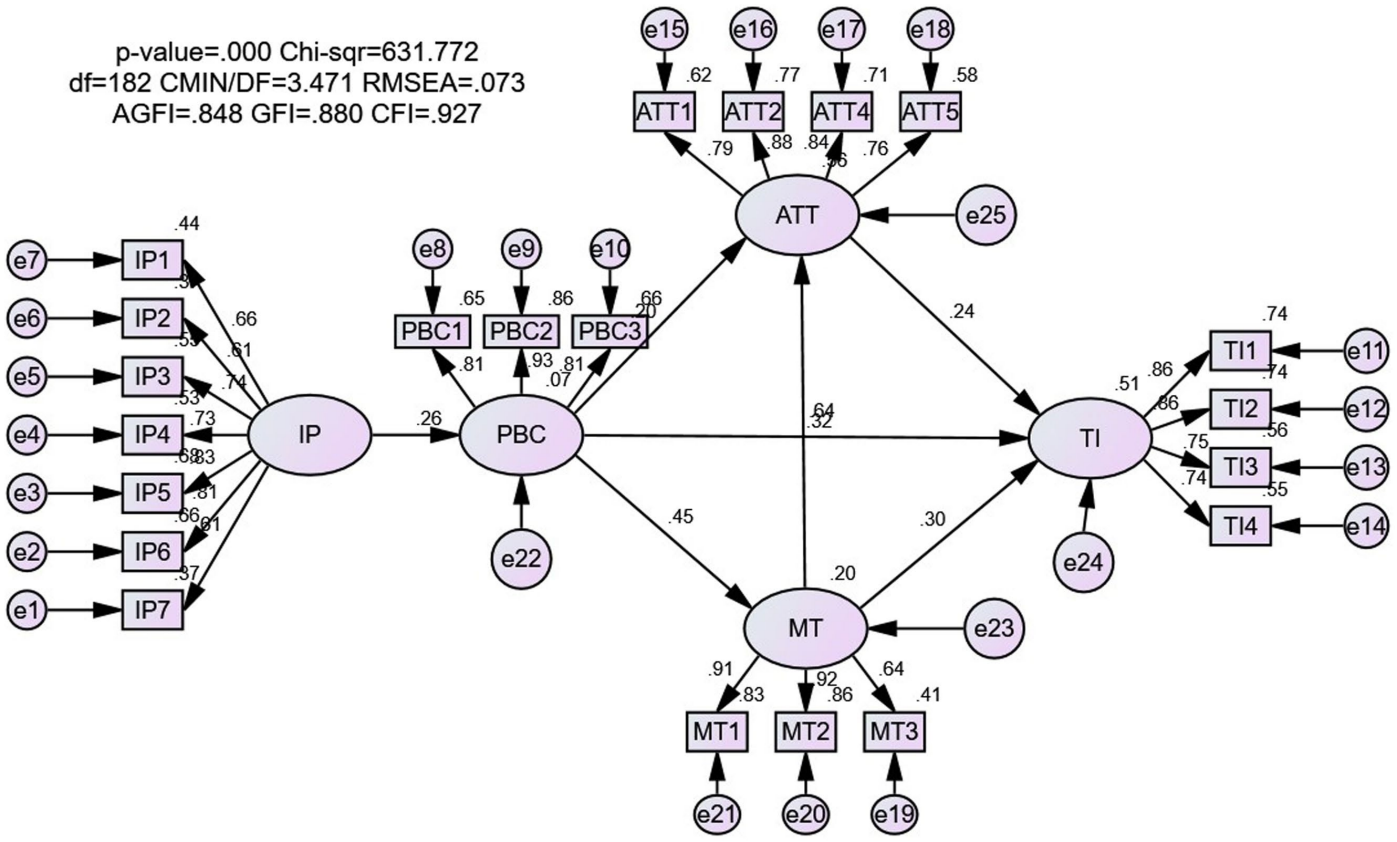
Measurement and structural model analysis.

As shown in Figure 2 and Table 6, the path coefficients β of the seven hypotheses were all greater than 0.20, and the values of *p* were all less than 0.001, reaching a significant level and indicating that the seven hypotheses were all verified.

**Table 6:** Outcomes of structural equation modeling analysis (*N* = 470).

Hypothesis	Path	Path coefficient (*β*)	S.E.	C.R.	P	Supported?
H1	IP --- > PBC	0.260	0.081	4.900	***	Yes
H2	PBC --- > ATT	0.200	0.040	4.559	***	Yes
H3	PBC --- > TI	0.320	0.052	6.778	***	Yes
H4	PBC --- > MT	0.450	0.044	8.266	***	Yes
H5	ATT --- > TI	0.240	0.079	3.680	***	Yes
H6 H7	MT --- > TI MT --- > ATT	0.300 0.640	0.088 0.068	4.583 10.632	*** ***	Yes Yes

*** *P < 0.001.*

### Analysis of the Mediating Effect

The bootstrap method and Sobel Z test were used to test the mediating effect (Table 7). The indirect effect of perceptions of positive COVID-19 information on travel intention through perceived behavioral control was 0.108 (SE = 0.027, *Z* = 4 > 1.96, *p* = 0.000 < 0.05). Further, the upper and lower limits of bias-corrected 95% CI and percentile 95% CI did not contain 0; therefore, the indirect effect was confirmed. The indirect effect of perceptions of positive COVID-19 information on travel intention through perceived behavioral control and travel attitude was 0.016 (SE = 0.008, Z = 2 > 1.96, *p* = 0.007 < 0.05); through perceived behavioral control and travel motivation was 0.045 (SE = 0.020, *z* = 2.25 > 1.96, *p* = 0.000 < 0.05); and through perceived behavioral control, travel motivation, and travel attitude was 0.023 (SE = 0.001, *Z* = 2.3 > 1.96, *p* < 0.05).

**Table 7:** Intermediary effect test table.

Relationships	Point estimate	Product of coefficient		Bootstrap 1,000 times 95%CI				
				Bias-corrected		Percentile		P
		SE	Z	Lower	Upper	Lower	Upper	
Indirect effects								
IP → PBC → TI	0.108	0.027	4	0.061	0.172	0.058	0.167	0.000
IP → PBC → ATT → TI	0.016	0.008	2	0.005	0.040	0.003	0.035	0.007
IP → PBC → MT → TI	0.045	0.020	2.25	0.017	0.096	0.015	0.091	0.000
IP → PBC → MT → ATT → TI	0.023	0.010	2.3	0.008	0.051	0.006	0.046	0.007
Total Indirect Effects	0.192	0.046	4.17	0.107	0.290	0.106	0.286	0.000
Contrasts								
PBC vs. ATT	0.092	0.027	3.41	0.046	0.156	0.043	0.151	0.000
PBC vs. MT	0.063	0.026	2.42	0.018	0.122	0.013	0.114	0.014
PBC vs. MT-ATT	0.085	0.027	3.15	0.041	0.148	0.037	0.143	0.000
ATT vs. MT	−0.029	0.022	−1.32	−0.083	0.005	−0.080	0.007	0.096
ATT vs. MT-ATT	−0.007	0.007	−1	−0.030	0.002	−0.025	0.005	0.266
MT vs. MT-ATT	0.022	0.022	1	−0.013	0.076	−0.014	0.071	0.203

Among the indirect effects, perceptions of positive COVID-19 information had the largest mediating effect on travel intention through perceived behavioral control. However, the direct effect in this study was not significant, indicating that perceptions of positive COVID-19 information had a complete mediating effect on travel intention through perceived behavioral control, travel attitude, and travel motivation.

## Discussion and Conclusion

Based on the Theory of Planned Behavior and the background of the COVID-19 pandemic, this study reveals that perceptions of positive COVID-19 information positively impacted Chinese university students’ perceived behavioral control, while their perceived behavioral control had a positive impact on their travel attitudes, motivations, and intentions. This finding is consistent with those of previous studies that have demonstrated the negative impact of environmental risks on travel intention (Rittichainuwat and Chakraborty, 2009; Quintal et al., 2010) and the role of positive information perception in stimulating tourists’ travel intentions (Kuo et al., 2015; Choufany, 2020; Ivanova et al., 2021). The more positive COVID-19 information tourists obtained from various sources, the more they perceived they could control their travel-related behavior. On one hand, positive news related to COVID-19 decreased the university students’ risk perceptions, self-protection motivations, and fear of traveling (Qiao et al., 2021). On the other, the re-opening of transportation, visitor attractions, and tourism services assured the feasibility of traveling (Puca et al., 2020).

Positive COVID-19 information perceptions had no direct influence but did display a complete mediating effect on travel intention through perceived behavioral control, attitude, and motivation. This result is in accordance with Zhu and Deng (2020), who revealed that available information indirectly influenced people’s intention to travel during the COVID-19 pandemic. Moreover, this study also confirmed the mediating effect of perceived behavioral control on travel intention (Sánchez-Cañizares et al., 2021). Scholars have previously verified that negative information causes emotional anxiety and fear, thus lowering perceived behavioral control and further lowering travel motivation and intention (Bae and Chang, 2021; Zheng et al., 2021). In contrast, our study confirmed the positive role that positive COVID-19 information plays in enhancing travel motivation and intention, echoing Tavitiyaman and Qu (2013).

Our finding contradicts with Li et al.’s (2021) conclusion that people’s travel intentions would still be negatively influenced 6 months or even longer after the COVID-19 pandemic was controlled. Lengthy lockdowns lead to both physical and psychological needs for leisure and escape. As long as the COVID-19 pandemic is under control, more positive information is perceived, and people’s perceived behavioral control is enhanced. This leads to positive attitudes toward travel, the release of suppressed travel motivation, and, ultimately, the increase of travel intention. This was particularly obvious among the Chinese university students who had been forced to stay at home and take classes online for a long time. They paid special attention to any positive information regarding the COVID-19 pandemic and the tourism industry. With increased perceived behavioral control, they generated positive travel attitudes, motivations, and intentions, which can transform into actual travel once other preconditions (e.g., time and money) are met. At present, the pandemic is under control in China and some other countries, and the tourism industry is gradually recovering. We believe that COVID-19 will be controlled in the near future as governments adopt relevant measures and the gradual use of vaccines. According to the results of this study, with perceptions of more positive information about the pandemic’s progress, people’s travel intention will be increased.

### Implications

We investigated the travel motivation and intention of Chinese university students during the tourism industry’s recovery from the effects of the COVID-19 pandemic, adding the variable of positive information perceptions to the relationship between the pandemic and tourism for the first time. First, this study contributes to our understanding of the potential recovery of the tourism industry during and after the COVID-19 pandemic. While previous studies mainly focused on the dark side of the COVID-19 pandemic on travel motivation and intention, our study fills the knowledge gap of the bright side of positive COVID-19 information. The COVID-19 pandemic has the potential to alter the ways in which tourists assess risks and form their risk perception (Jahari et al., 2021). Different from the general positive information perception (e.g., good service quality), COVID-19 positive information perception relates to travelers’ first priority health and safety. It decreases travelers’ emotional anxiety and fear for pandemic, which is the decisive factor of travel motivation and travel intention. Second, our study also enriches the research on university students’ travel motivations and intentions in terms of the impacts of risk and information perceptions. Previous studies have confirmed university students’ travel intentions would decrease in face of risks (Hartjes et al., 2009; Kim et al., 2012). The findings in this study reveal that university students are risk-takers and crisis-resistant tourists (Hajibaba et al., 2015) when the information they perceive is positive. Third, it extends the application of the Theory of Planned Behavior in the tourism research by adding the dimension of positive information perceptions. It asserts the effectiveness of the Theory of Planned Behavior in predicting tourists’ intention and behavior.

This study also offers some practical implications. According to the results, perceptions of positive COVID-19 information mainly affect travel intention through perceived behavioral control. Therefore, tourism operators should take measures to increase undergraduate students’ perceived behavioral control, thereby increasing their travel intentions. This includes delivering various positive information to undergraduate students, including the positive COVID-19 situation at destinations, the measures taken in these destinations to prevent infection, the tourism services provided, and so on. Corresponding measures should be taken to strengthen the use of technology from the perspective of information perception, especially in this special period, to ensure the wide circulation and authenticity of information. Some smart phone applications which are popular among Chinese undergraduate students (e.g., WeChat and Douyin) should be paid special attention. Tourism managers can spread relevant security information about the COVID-19 pandemic prevention measures that potential tourists may pay attention to. During travel, tourism operators should also employ measures to reduce the risks of tourists’ exposure to infection, such as improving sanitation and disinfection services, strictly controlling the number of tourists entering visitor attractions, and offering different viewing routes designed to divert tourists and avoid the formation of crowds.

### Limitations and Future Research

Due to the time limitation and impacts of the COVID-19 pandemic, only 470 valid samples were collected online. Future research should expand the scope of this study’s survey and the sample size and improve the quality of the data collection by distributing questionnaires both online and offline. Although the questionnaire showed good reliability and validity within the acceptable range, this study only explored travel motivation and intention based on the components of the theory of planned behavior, while overlooking other influencing factors. Future research should include these other influencing factors, based on other theories, to investigate the impact of positive COVID-19 information perceptions on travel motivation and intention.

Furthermore, the survey was limited to China, where the pandemic has been controlled to a large extent. Consequently, most respondents were from low-risk areas, and many of them had recently undertaken tourism activities. The results may differ in countries where the pandemic situation is still critical. Moreover, as Kaczmarek et al. (2021) noted, collectivist countries have tended to cope better with the pandemic, and China is a good example of this. Government control has played a significant role in tourism recovery during the pandemic (Fong et al., 2021), and their trust in the government may increase Chinese tourists’ travel intentions by decreasing their fear (Zheng et al., 2021). Future research should be undertaken in other more individualist countries to determine if the results differ. In addition, the sample of this study is Chinese university students who are “allocentric” and rely on their parents’ economic support for travel, which limits its findings to be generalized. The respondent sample should be extended to other groups, such as the elderly, to gain a comprehensive understanding of the relationship between positive COVID-19 information perceptions and travel intention.

## Data Availability Statement

The raw data supporting the conclusions of this article will be made available by the authors, without undue reservation.

## Ethics Statement

Ethical review and approval were not required for the study of human participants in accordance with the local legislation and institutional requirements. Written informed consent from the participants was not required to participate in this study in accordance with the national legislation and the institutional requirements.

## Author Contributions

All authors listed have made a substantial, direct, and intellectual contribution to the work and approved it for publication.

## Funding

This paper is supported by the National Social Science Fund projects “The research on intelligent elderly care service mode of sports and medicine integration” (no. 21XTY006).

## Conflict of Interest

The authors declare that the research was conducted in the absence of any commercial or financial relationships that could be construed as a potential conflict of interest.

## Publisher’s Note

All claims expressed in this article are solely those of the authors and do not necessarily represent those of their affiliated organizations, or those of the publisher, the editors and the reviewers. Any product that may be evaluated in this article, or claim that may be made by its manufacturer, is not guaranteed or endorsed by the publisher.

## Footnotes

^1^ www.wjx.cn
